# A route planning for oil sample transportation based on improved A* algorithm

**DOI:** 10.1038/s41598-023-49266-z

**Published:** 2023-12-12

**Authors:** Yingjun Sang, Xianyan Chen, Quanyu Chen, Jinglei Tao, Yuanyuan Fan

**Affiliations:** 1 Faculty of Automation, Huaiyin Institute of Technology, Huaian, 223003 China; 2 Faculty of Mathematics and Physics, Huaiyin Institute of Technology, Huaian, 223003 China

**Keywords:** Chemical engineering, Engineering, Mathematics and computing

## Abstract

The traditional A* algorithm suffers from issues such as sharp turning points in the path, weak directional guidance during the search, and a large number of computed nodes. To address these problems, a modified approach called the Directional Search A* algorithm along with a path smoothing technique has been proposed. Firstly, the Directional Search A* algorithm introduces an angle constraint condition through the evaluation function. By converting sharp turns into obtuse angles, the path turning points become smoother. This approach reduces the occurrence of sharp turns in the path, resulting in improved path smoothness. Secondly, the algorithm enhances the distance function to strengthen the directional guidance during the path search. By optimizing the distance function, the algorithm tends to prefer directions that lead towards the target, which helps reduce the search space and shorten the overall path planning time. Additionally, the algorithm removes redundant nodes along the path, resulting in a more concise path representation. Lastly, the algorithm proposes an improved step size adjustment method to optimize the number of path nodes obtained. By appropriately adjusting the step size, the algorithm further reduces the number of nodes, leading to improved path planning efficiency. By applying these methods together, the Directional Search A* algorithm effectively addresses the limitations of the traditional A* algorithm and produces smoother and more efficient path planning results. Simulation experiments comparing the modified A* algorithm with the traditional A* algorithm were conducted using MATLAB. The experimental results demonstrate that the improved A* algorithm can generate shorter paths, with reduced planning time and smoother trajectories. This indicates that the Directional Search A* algorithm, incorporating the angle constraint condition in the evaluation function and the direction-guided strategy, outperforms the traditional A* algorithm in path planning and provides better solutions to the existing issues.

## Introduction

The optimization of robot path planning algorithms is primarily aimed at improving the performance and efficiency of robots in practical applications. By optimizing the path planning algorithm, robots can reach their target locations faster, thereby enhancing the overall efficiency and accuracy of the motion process^1^. Additionally, optimizing path planning algorithms can assist robots in selecting the shortest and most economical paths when performing tasks, thus reducing the cost of robot movements. Furthermore, optimizing path planning algorithms can help robots avoid collisions with obstacles and hazardous areas, thereby enhancing robot safety^2^. By optimizing robot path planning algorithms, we can better meet the requirements of real-world applications and drive the development and application of robot technology.

The first-generation industrial robots used a coordinate transformation-based approach to control the execution trajectory^3^. This method performed well for straightforward tasks but encountered challenges in ensuring precision and stability within intricate environments and tasks. To tackle this predicament, researchers commenced their exploration of robot path planning algorithms. Initially, robot path planning algorithms primarily relied on geometric models to calculate the robotic motion trajectory, such as straight-line and circular arc methods^4,5^. These algorithms could simply describe the robot’s motion path but proved ineffective in handling complex objects or spatial obstacles. Over the next few decades, an increasing number of researchers delved into advanced algorithms to adapt to the complexity of work environments. Techniques like graph theory, search algorithms, and artificial intelligence were used for path planning. Algorithms such as A*, Dijkstra’s algorithm, Rapidly-exploring Random Trees (RRT), and Probabilistic Roadmaps (PRM) gained widespread application^6^. In recent years, with the development of deep learning technology, artificial neural networks have been introduced to the field of robot path planning. Training neural networks enables more intelligent and efficient path planning^7^. These algorithms not only improve the speed and accuracy of robot path planning but also enable adaptive adjustment of path planning strategies within complex environments, allowing robots to better adapt to various working conditions.

The traditional A* algorithm commonly uses Manhattan distance and Euclidean distance^8^. The Manhattan distance is obtained by summing the absolute differences in x and y values between the expanding node and the target node, which can result in an overestimation of h(n). On the other hand, the Euclidean distance is the straight-line distance from the expanding node to the target node, leading to an underestimation of h(n). Li et al.^9^ adopted the second-order Bessel curve function as the heuristic function to meet the requirements of smooth path planning. Although the resulting path is smoother, it tends to approximate the second-order Bézier curve and may not align well with the actual scene. Therefore, adding corner constraints can not only maintain stable driving in path planning, but also adjust the path reasonably according to the actual environment. This ensures that the planned path is not only smooth but also compatible with the real-world scenario.

With the advancement of deep learning technology, an increasing number of researchers are turning to neural networks for robot path planning^10^. These methods require less manual design and can directly learn appropriate planning strategies based on input data, resulting in higher accuracy and efficiency. Integrated with environment perception, path planning that leverages neural networks involves real-time monitoring and analysis of the surroundings, enabling more intelligent decision-making in path planning^11^. However, neural networks rely heavily on training data to build models. This data needs to encompass various scenarios and complex traffic situations. If the collected data is insufficient or limited, the predictive capability of the neural network will be greatly affected, leading to unreliable path planning results^12^. Moreover, the training data for neural networks often comes from a series of classical path planning algorithms. To obtain better paths, improvements must be made to these classical algorithms to generate superior training data. Therefore, it is necessary to enhance the A* algorithm to achieve optimal path planning results.

The main research content of this paper can be summarized as follows:

- Introduction of Angle Constraint in the Heuristic Function: The manuscript posits the integration of an angle constraint into the heuristic function, with the intent of refining the path, diminishing path length, curtailing path search duration, and enhancing obstacle avoidance proficiency. By conscientiously considering the angles between consecutive path segments, the algorithm aspires to engender pathways that are both more efficient and more attainable.
- Path Search Direction Guidance Strategy: The paper introduces a path search direction guidance strategy to help the algorithm quickly determine the search direction, avoiding repetitive searching of feasible points due to obstacles. This strategy not only amplifies the efficiency of the search but also prevents the algorithm from getting trapped in local optima or deadlock situations, thus ensuring the possibility of obtaining globally optimal solutions.
- Improved Path Search Step Size: The paper highlights the importance of adjusting the path search step size to enhance search efficiency, save computational resources, improve search accuracy, and make the algorithm more suitable for complex environments. By dynamically adapting the step size based on the local environment, the algorithm can effectively navigate through obstacles and find optimal paths.

The remaining sections of the paper are structured as follows: Section “Background” provides an overview of the research context and background. Section “Optimize A * algorithm” presents the theoretical foundations and methodologies of the improved A* algorithm. Section “Simulation analysis” describes the simulation experiments conducted to validate the feasibility and effectiveness of the proposed algorithm enhancements. Section “Conclusion” discusses potential future research directions and offers recommendations for further improvements.

## Background

In the petrochemical industry, petroleum testing plays a crucial role in detecting and analyzing the physical and chemical characteristics of petroleum and its derivatives. It helps in formulating production and processing strategies to ensure product quality and safety. Petroleum testing often involves multiple testing parameters, which may have complex interdependence and specific sequential requirements. Additionally, petroleum samples need to be transported to multiple destinations. Therefore, path planning for robots in petroleum testing becomes necessary to determine the optimal transport routes, aiming to enhance the efficiency and accuracy of petroleum testing. With the continuous development of computer technology and artificial intelligence, robot path planning techniques can now be automated and intelligent through the use of models and algorithms^13^. This brings more convenience and benefits to industrial production.

Currently, petroleum samples in chemical plants are still collected manually. However, due to the presence of hazardous areas and hard-to-reach locations in petrochemical plants, manual sampling is difficult, time-consuming, and poses high risk. Therefore, in order to enhance the level of intelligence in the petroleum testing process, reduce labor intensity, and improve efficiency, we propose using robots to replace manual labor in the transportation of petroleum samples^14^. Additionally, during robot path planning, we will consider the environmental characteristics of the petrochemical plant. This approach allows the transportation robot to play a crucial role in petroleum sampling.

Many scholars have conducted research on optimizing robot path planning. Susan Sorensen et al. focused on the multi-track routing and scheduling optimization problem for in-orbit refueling space robots^15^. The objective of this problem is to enable a group of highly maneuverable and refuelable space robots to complete a set of tasks on space orbits through optimal path planning and task allocation. To address this problem, the researchers adopted a mixed-integer linear programming approach and evaluated the quality of solutions by maximizing the weighted number of completed tasks. Through case studies and the use of data from satellites operating in different Earth orbits, the researchers demonstrated algorithms for constructing networks and models. They also proved that considering strategies involving multiple orbits is beneficial when it comes to robot motion, the number of refueling stations, and task dependencies. Overall, their research provides insights into optimizing path planning and scheduling for space robots performing complex tasks such as in-orbit refueling. Li et al.^16^ proposed an improved Iterative Greedy (IIG) algorithm for the multi-factory robot scheduling problem. They primarily focused on the distributed flow shop problem with order constraints, where jobs of the same production order need to be assigned to the same factory. The algorithm also takes into account deteriorating time limits to minimize job completion time in the factories. Lu et al. proposed a multi-AGV scheduling method considering allocation rules for automatic guided vehicles (AGVs) due to the increase in workload leading to conflicts and deadlocks, and designed a phase conflict avoidance method to generate conflict-free working routes for AGVs in automated warehouses with multiple workstations^17^. Nguyen et al. proposed a path optimization method for the lower limit of logistics distribution vehicle capacity in their research, which can help the company achieve a lot of operating cost savings^18^. By optimizing the route planning of vehicles, this method can ensure that the loading capacity of each vehicle is not lower than the set lower limit, thereby improving transportation efficiency and resource utilization. The application of this method can help logistics companies to arrange delivery vehicles more effectively and reduce operating costs.

Path planning constitutes a formidable challenge within the realm of oil sample transportation, owing to its time-intensive and computationally demanding nature in practical implementations. Ensuring efficiency and quality remains a formidable task. Therefore, it is of great significance to realize high-quality path planning technology for oil sample transportation^19,20^. Although there are a large number of literatures for reference, the current oil sampling is mainly realized manually, and the research on the path planning of the robot in the process of oil sample transportation is still relatively weak.

**Figure 1:**
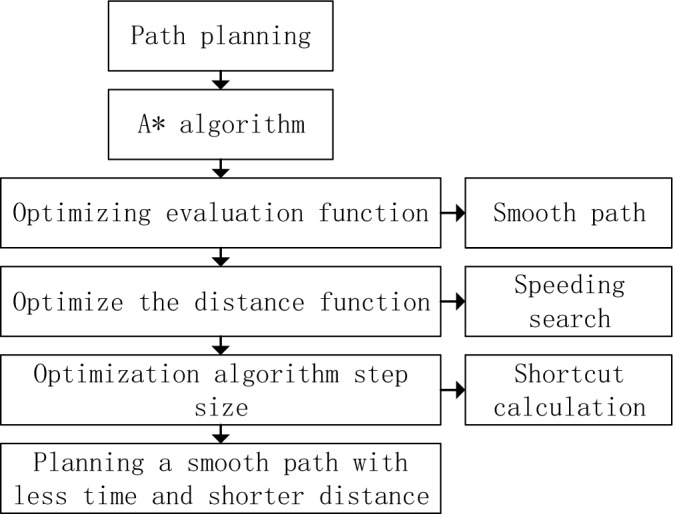
Block diagram of algorithm optimization.

This paper proposes an A* algorithm for oil sample transportation path optimization. The algorithm first introduces the angle constraint condition to make the turning of the path smoother. Secondly, the distance function of A* algorithm is improved to enhance the direction of path search and shorten the path planning time. Finally, the path-finding step size of the algorithm is adjusted to reduce the amount of calculation and improve the efficiency of oil sample transportation. These improvements have raised the application of A* algorithm in oil sample transportation to a new level. The algorithm optimization block diagram proposed in this paper is shown in Fig. 1, which can get a path with short planning time, short path length and high smoothness. These optimization schemes make the A* algorithm more applicable and efficient in the transportation of oil samples.

## Optimize A * algorithm

The A* algorithm is a heuristic path-finding algorithm used to compute optimal paths in a static two-dimensional space. It combines the global path-planning algorithm Dijkstra's algorithm with the heuristic best-first search algorithm BFS, using a cost evaluation function as the evaluation metric, where a smaller function value indicates a more optimal path node. The algorithm forms the optimal path by progressively selecting the most optimal nodes. The cost evaluation function is as follows:

1 $$ F(n) = h(n) + g(n) $$

where *F*(*n*) is the cost evaluation function of the current position *n*, *h*(*n*) is the actual cost of the robot from the initial position to the current position *n*, and *g*(*n*) is the estimated cost of the robot from the current position *n* to the target position. The size of *F*(*n*) will directly determine the selection of the optimal path node, thus affecting the path planning effect of A * algorithm. The choice of *g*(*n*) will also indirectly affect the path planning efficiency of A * algorithm. The closer the value is to the actual value, the higher the chance of successful path search. In the grid map environment, there are three distance models:

1. Manhattan distance

The distance between two nodes is represented by the sum of the distances in the horizontal and vertical directions. It is calculated as the absolute difference between the x-coordinates plus the absolute difference between the y-coordinates of the two nodes.

2 $$ D_{Manhat\tan } (i,j) = \left| {x_{2} - x_{1} } \right| + \left| {y_{2} - y{}_{1}} \right| $$

where “x” represents the horizontal coordinate and “*y*” represents the vertical coordinate. “1” and “2” represent two different location nodes. When considering only the four directions of movement for the robot (up, down, left, right), the Manhattan distance can be used.

(2) Chebyshev Distance:

The distance between two nodes is represented by the maximum of the absolute difference between their x-coordinates and the absolute difference between their y-coordinates. It calculates the maximum vertical or horizontal moves required to reach one node from the other.

3 $$ D_{Chebyshev} (i,j) = \max (\left| {x_{2} - x_{1} } \right|,\left| {y_{2} - y_{1} } \right|) $$

When considering the robot's movement in all eight directions (diagonal, vertical, and horizontal), the Chebyshev distance can be used.

(3) Euclidean Distance:

The distance between two nodes is represented by the square root of the sum of the squares of the differences between their x-coordinates and y-coordinates. It calculates the straight-line distance between the two points in a two-dimensional plane.

4 $$ D_{Euclidean} (i,j) = \sqrt {\left( {x_{2} - x_{1} } \right)^{2} + \left( {y_{2} - y_{1} } \right)^{2} } $$

When the robot can move in any direction randomly, the Euclidean distance can be used.

A* algorithm is widely used in the field of robot path planning. However, when applying this algorithm to petroleum laboratory scenarios, there may be some issues with the generated paths, such as excessive curvature, long planning time, and weak directionality in path searching. Therefore, it is necessary to optimize this algorithm to meet the specific requirements of petroleum sample transportation.

### Corner constraint

In petroleum sample transportation, the original A* algorithm's heuristic function only considers distance cost constraints without taking into account the turning cost constraints during the path. This can lead to issues where the generated path has excessively sharp turns, which can impact the effectiveness of the vehicle's movement. In segments with right-angle turns, the vehicle needs to decelerate before accelerating to pass through. The sharper the turn, the longer the time required for the vehicle to complete the turn, reducing overall efficiency and safety. Additionally, due to the limitations of the vehicle’s steering mechanism, it cannot perform excessively sharp turns. Therefore, it is necessary to optimize the path planning of the A* algorithm to alleviate this situation. By considering special considerations for right-angle turns in the path, the path can be made smoother, thereby improving efficiency and safety in petroleum sample transportation.

5 $$ F(n) = h(n) + g(n),\;n \in p(\theta ) $$

6 $$ h(n) = historic(a,b) = \sqrt {sum(a - b)^{2} } $$

7 $$ g(n) = heuristic(x,goal) = \sqrt {sum(x - goal)^{2} } $$

8 $$ p(\theta ) = \left\{ {\begin{array}{*{20}c} {135^\circ ,turn} \\ {0^\circ ,straight\;forward} \\ \end{array} } \right. $$

where *F*(*n*) is the estimated cost of the best path from the initial node to the end point; *h*(*n*) is the estimated cost from the initial node to the node *n*; *g*(*n*) is the estimated cost from the node *n* to the target node; *p*(*θ*) is the corner constraint condition function, and *θ* is the number of internal angles formed by three consecutive nodes.

**Figure 2:**
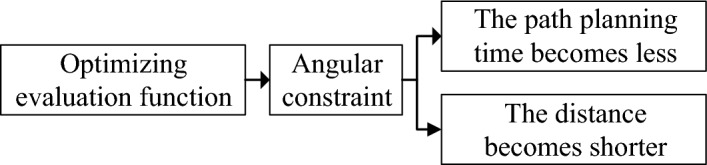
Block diagram of evaluation function optimization.

By incorporating angle constraints into the heuristic function of the A* algorithm, we can optimize the path planning to achieve obtuse-angle turns, as shown in Fig. 2. This optimization helps reduce the turning time and cost for the vehicle while ensuring smoother and more efficient paths. With this optimization, the path planning in petroleum sample transportation can better adapt to the vehicle's steering mechanism and driving requirements, thereby improving overall efficiency and safety.

**Figure 3:**
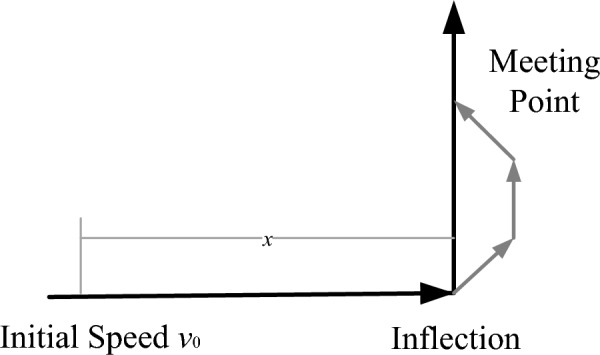
Improved A* algorithm bending calculation chart.

At the right-angle turn depicted in Fig. 3, the vehicle needs to undergo uniform deceleration. As it approaches the corner, the vehicle gradually reduces its speed to bring it to a complete stop before initiating the turn, ensuring a smooth and safe passage through that segment. This process of uniform deceleration takes into consideration factors such as the vehicle’s turning radius, velocity, and steering inertia to ensure smooth and safe travel. Let *v*_0_ be the initial speed of the trolley,* x* be the braking distance, and *t*_1_ be the braking time.

9 $$ v_{0}^{2} = 2ax $$

10 $$ x = \frac{1}{2}at_{1}^{2} $$

The braking time of the car is:

11 $$ t_{1} = \frac{2x}{{v_{0} }} $$

When *x* > 3, Starting from the turning point, the vehicle undergoes uniform acceleration until it reaches its original speed, and then continues to travel at a constant velocity until reaching the intersection point where the improved path intersects with the original path. The time it takes for the vehicle to accelerate from the turning point until it reaches its original speed is denoted as *t*_2_. This point where the improved path intersects with the original path is called the intersection point. Let *t*_2_ the time to accelerate from the inflection point to *v*_0._

12 $$ t_{2} = t_{1} $$

13 $$ t_{before} = t_{1} + t_{2} = \frac{4x}{{v_{0} }} $$

14 $$ t_{later} = \frac{2x + 2\sqrt 2 - 2}{{v_{0} }} $$

$$ {\rm{get }} t_{before} > t_{later}$$.

When *x* = 3, From the turning point, the vehicle undergoes uniform acceleration until it reaches the intersection point, and then continues to travel at a constant velocity.

15 $$ t_{before} = t_{1} + t_{2} = \frac{12}{{v_{0} }} $$

16 $$ t_{later} = \frac{x + 2\sqrt 2 + 1}{{v_{0} }} = \frac{4 + 2\sqrt 2 }{{v_{0} }} $$

$${\text{get }}\, t_{before} > t_{later}$$.

When *x* < 3, starting from the inflection point, first accelerate uniformly to a velocity of *v*_0_, and then move uniformly to the junction.

17 $$ t_{before} = t_{1} + t_{2} + t_{e} = \frac{3x + 3}{{v_{0} }} $$

18 $$ t_{later} = \frac{x + 2\sqrt 2 + 1}{{v_{0} }} $$

When $$\sqrt 2 - 1 < x < 3$$,

$$ t_{before} > t_{later} $$

When $$0 < x < \sqrt 2 - 1$$

$$ t_{before} < t_{later} $$

In summary, when the trolley braking distance is greater than (√2) − 1, (Let the side length of the small square be 1), The improved A* algorithm takes less time at the turning point. This is because the improved algorithm considers the angle constraints in the path and optimizes the heuristic function, allowing the vehicle to make smoother turns at the turning point. This helps to avoid sharp turns, unnecessary deceleration, and acceleration issues, thus reducing the travel time of the vehicle on that segment. It improves the efficiency and safety of the entire driving process.

### Search direction guidance strategy

During the path searching process, although the optimized A* algorithm can generate paths that comply with the robot's driving rules, it lacks strong directional guidance, resulting in the need to exclude a large number of redundant nodes during the search and thus reducing the efficiency of path searching. Therefore, an optimization strategy can be applied to enhance the directional guidance. This strategy determines the direction of path searching by considering the order and relationships of test items, aiming to reduce the search space and improve the efficiency and accuracy of path planning. Additionally, improvements can also be made to the Euclidean distance formula used in the algorithm.

19 $$ g^{*} = heuristic(x,goal) = \sqrt {sum(x - goal)^{3} } $$

Adopting a directional guidance strategy to optimize the A* algorithm can significantly enhance the directionality and efficiency of path planning. Specifically, by incorporating directional information in distance estimation, the distances can be more accurately estimated. Additionally, the direction of path searching can be determined based on the sequential order of petroleum assay projects, resulting in a significant improvement in both accuracy and efficiency of path planning.

**Figure 4:**
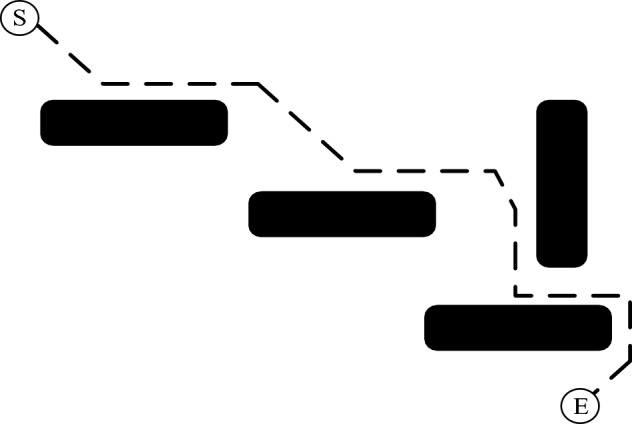
Schematic diagram of search direction.

According to Fig. 4, the black squares represent obstacles, and the black dashed line represents the optimal path planned from the start point (S) to the end point (E). The improved directional guidance A* algorithm, during the process of path searching, always searches in the direction towards the end point without choosing a direction that is farther from the end point just because it bypasses the obstacles with a shorter distance. This enhances the directionality of the algorithm's path searching and enables it to quickly find the optimal path nodes. When the algorithm searches for the optimal path, if there are no vertically oriented horizontal obstacles encountered, the nodes explored during the path search will be the optimal path nodes. The improved algorithm exhibits strong directionality in path searching. However, if there are vertically oriented horizontal obstacles, the improved algorithm will expand on both sides along the surface of the horizontal obstacle, searching for the first path node that can pass through the obstacle. The node that is first to pass through the obstacle becomes the optimal path, allowing the algorithm to bypass the horizontal obstacle and continue planning the path towards the target point.

### Path search step optimization

Reasonable adjustment of path search step can effectively improve the search efficiency. Increasing the search step can make the algorithm find the target node faster, and reducing the search step can refine the search range. Reasonably adjust the search step size to avoid falling into useless search areas during the path search process and improve the path search efficiency. In addition, the path search step size also affects the running time and space consumption of the search algorithm. Properly reducing the search step size can not only save computing resources, but also avoid problems such as crashes or running timeouts due to excessive computation. The path search step also has a certain influence on the search accuracy of the algorithm. If a smaller search step is used, the search algorithm can search the target area more carefully, improve the search accuracy, and avoid bypassing the target. Path search in complex environments often requires different search steps. For example, in flat areas, a larger step size can be used to improve the search efficiency, while in narrow and complex areas such as rugged mountain roads and buildings, a smaller step size is needed to ensure that the optimal path is found.

A* algorithm introduces Open and Close tables to store nodes in search. The Open table stores all the generated but not yet examined nodes, while the Close table records the visited nodes.A* The algorithm is to put each adjacent node into the Open table during the operation process, and find the optimal path step by step according to the small grid of the raster map during path planning, in which it is necessary to calculate the path evaluation cost of the continuous node to find the optimal path node, and the storage amount of the Open table increases, which increases the calculation amount of the algorithm.

In order to improve the running efficiency of the A* algorithm, the continuous nodes stored in the Open table are improved to nodes with interval steps, which increases the span between path nodes, reduces the data storage of the Open table, and reduces the amount of algorithm calculation.

As shown in Table 1, the start position is assumed to be X_31_ and the end position is X_39_.

**Table 1:** Raster map representation.

X_11_	X_12_	X_13_	X_14_	X_15_	X_16_	X_17_	X_18_	X_19_
X_21_	X_22_	X_23_	X_24_	X_25_	X_26_	X_27_	X_28_	X_29_
X_31_	X_32_	X_33_	X_34_	X_35_	X_36_	X_37_	X_38_	X_39_
X_41_	X_42_	X_43_	X_44_	X_45_	X_46_	X_47_	X_48_	X_49_
X_51_	X_52_	X_53_	X_54_	X_55_	X_56_	X_57_	X_58_	X_59_

The sequential step path of the original A* algorithm is: X_31_–X_32_–X_33_–X_34_–X_35_–X_36_–X_37_–X_38_–X_39_.

The path planned by the A* algorithm spaced one step apart is X_31_–X_33_–X_35_–X_37_–X_39_.

The path planned by the A* algorithm separated by two steps is X_31_–X_34_–X_37_–X_39_.

The path planned by the A* algorithm with three steps apart is X_31_–X_35_–X_39_.

However, blindly pursuing the efficiency of path search will reduce the feasibility of the search path. The larger the path search interval, although the efficiency of the algorithm is improved, there may be collisions caused by missed obstacle avoidance ; the smaller the path search interval, the longer the algorithm planning time, but the path obtained by this planning is more feasible. Considering the path search efficiency and complete obstacle avoidance factors, the path search step size of the specific environment is adjusted to make the path optimal in terms of planning time and feasibility.

## Simulation analysis

**Figure 5:**
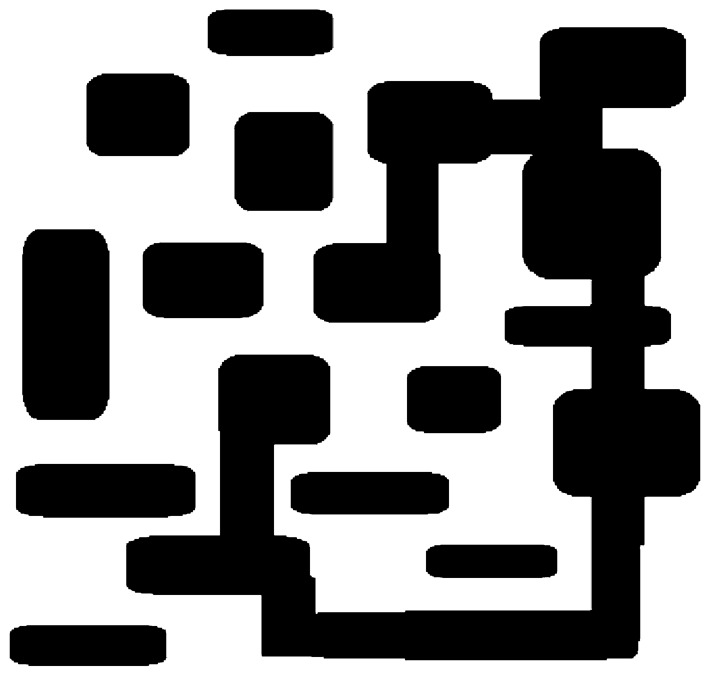
Top view of a petrochemical plant.

The experiment was conducted using MATLAB 2018a on a Lenovo computer with a frequency of 2.40 MHz and 4 GB of memory. In the experiment, a petroleum and chemical industry environment was constructed, measuring 100 dm × 100 dm. The environment was divided into square grids with a side length of 1 dm, and the equipment areas and hazardous areas were designated as obstacle regions. As shown in Fig. 5, the white part is the feasible region, and the black part is the obstacle which edge is expanded when constructing the map, leaving a safe distance around the obstacle, which is also indicated in the black area. The obstacle position in the experiment is setted randomly on the map.

### Comparison before and after corner constraint

**Figure 6:**
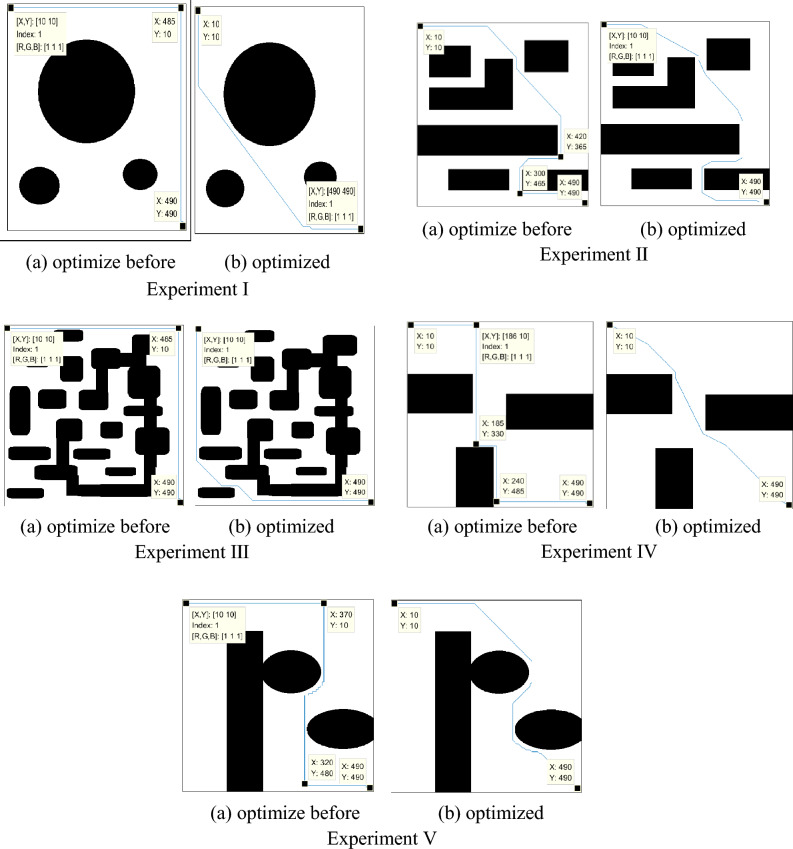
Simulation results before and after corner constraints.

The improved A* algorithm with an enhanced heuristic function plans the path as shown in Fig. 6. The black area represents obstacles, the white area represents the feasible region, and the blue line represents the optimal path obtained through planning. The starting point of the path is the top-left corner (10, 10), and the endpoint is the bottom-right corner (490, 490). From Fig. 6, it can be observed that, after optimizing the evaluation function, the path consists of obtuse-angle turns instead of right-angle turns, resulting in a smoother path. This path better aligns with the practical scenario of the petroleum sample transportation robot's movement.

To validate the impact of angle constraints in the evaluation function, considering that the path in the grid map follows the centers of the grid cells, the path turns can be either right-angle turns or diagonal turns. The bending angle is constrained to be either 180° or 135°, which means the path can only be a straight line or a diagonal direction and cannot have right-angle turns. Specifically, at each node, the connection between the current node, its previous parent node, and the next node to be traversed should form an internal angle of either 180° or 135° when selecting the next path point. A comparative experiment was conducted before and after the improvement, and the experimental results are shown in Fig. 6, with the corresponding data presented in Table 2.

**Table 2:** Experimental data before and after corner optimization.

Path planning	Algorithm	Plan the time 10^2^/s	The length of the path 10^2^/dm	Right angle number
Experiment I	Before optimization	2.140	9.600	1
	optimized	1.028	7.754	0
	optimization rate	51.96%	12.90%	1
Experiment II	Before optimization	1.621	1.045	2
	optimized	1.560	1.014	0
	optimization rate	3.76%	2.97%	1
Experiment III	Before optimization	1.225	9.600	3
	optimized	1.161	8.997	0
	optimization rate	5.22%	6.28%	1
Experiment IV	Before optimization	1.322	7.600	4
	optimized	0.672	5.901	0
	optimization rate	49.17%	22.36%	1
Experiment V	Before optimization	1.995	10.600	2
	optimized	1.726	10.193	0
	optimization rate	13.48%	3.84%	1

By conducting the five experiments mentioned above, it was found that both before and after the optimization, a path can be planned from the starting point to the destination. However, there were differences in the path planning time and the length of the planned path. From Table 2, it can be observed that the improved A* algorithm with the optimized heuristic function not only had shorter path planning time compared to the original A* algorithm but also had a shorter path length. This indicates that the A* algorithm, which replaces right-angle turns with obtuse-angle turns, is more efficient in path planning.

**Figure 7:**
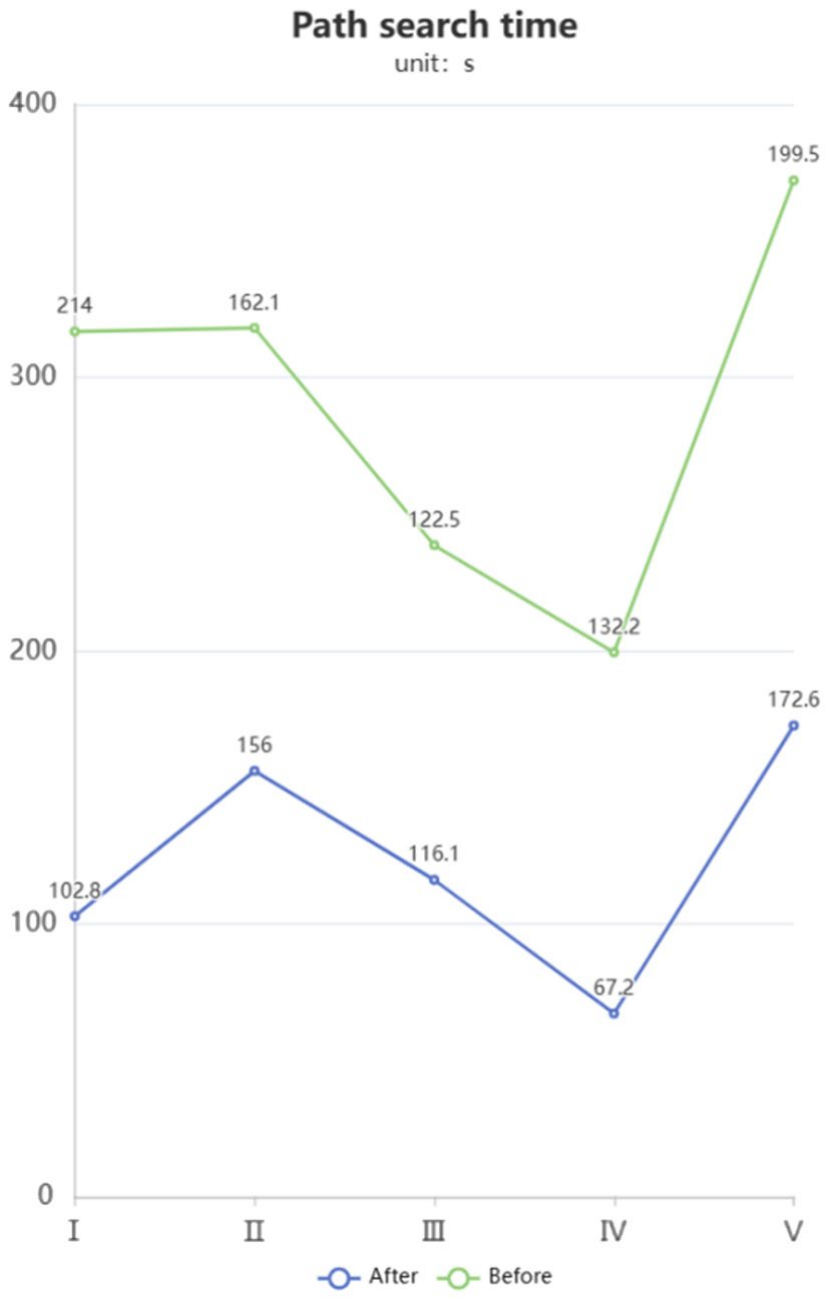
Line chart comparing the path planning time before and after corner optimization.

As shown in Fig. 7, the optimized A* algorithm significantly reduces the time required to plan a path from the starting point to the destination in each experiment. In different environments, even with the same starting and ending points, the optimized A* algorithm reduces the time required for path planning to varying degrees. Such optimization can save costs in path planning and create more benefits for petrochemical plants.

**Figure 8:**
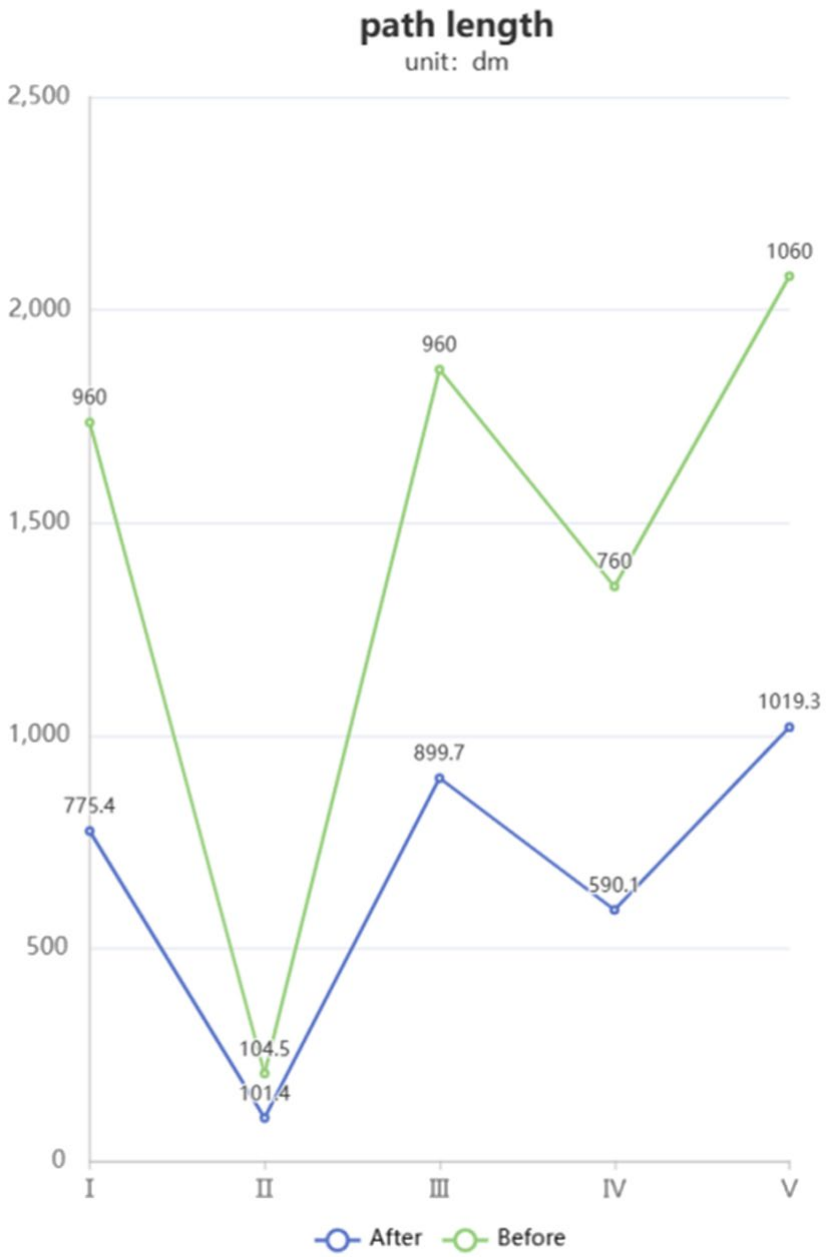
Comparison of path lengths before and after corner optimization.

Based on the results shown in Fig. 8, the optimized A* algorithm plans paths with shorter lengths compared to the paths planned by the original algorithm in each experiment. Whether in different environments or with the same starting and ending points, the optimized A* algorithm can significantly reduce the length of the planned paths. The paths after corner optimization are shorter, indicating that the improved algorithm is more effective in finding paths with better cost efficiency.

### Comparison before and after the introduction of direction-oriented strategy

To verify the impact of introducing the direction-guided strategy on path planning, we conducted comparative experiments while keeping other conditions constant. The original algorithm requires traversing all feasible nodes around the starting point and neighboring nodes of the path nodes in order to find the optimal path. This may result in excessive redundant node cost calculations. On the other hand, the optimized A* algorithm with the direction-guided strategy utilizes a heuristic function to guide the search, limiting the search direction to the path that is more likely to reach the target point. This reduces the computation of redundant nodes. Through the experimental results, it can be verified that the optimized A* algorithm exhibits higher efficiency and accuracy in path planning, enabling it to find a shorter optimal path.

**Figure 9:**
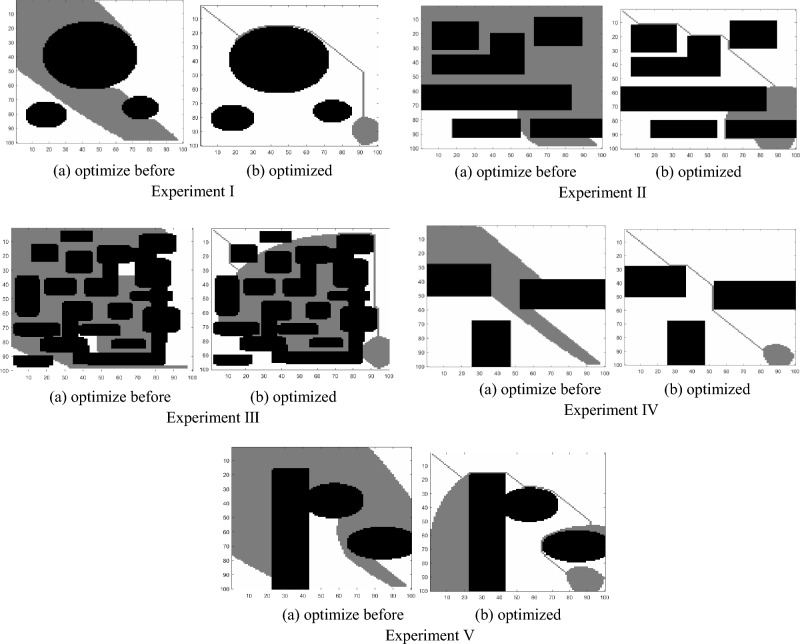
Path planning before and after the direction oriented strategy.

By comparing the original algorithm with the A* algorithm incorporating the direction-guided strategy through simulation experiments, we can observe the results depicted in Fig. 9. During the path planning process, the black region represents obstacles, the white region represents feasible areas, and the gray region represents the traversed areas during the search. The upper left position of each experimental map is the start point and the lower right position is the end point, finding an optimal path from the start position to the end position under the directional guidance strategy. Based on the experimental results, the following conclusions can be drawn: the A* algorithm with the direction-guided strategy exhibits higher efficiency in path planning and fewer redundant nodes compared to the original algorithm. This means that the optimized algorithm can find the optimal path more quickly. By restricting the search direction, the algorithm can accurately select the next expansion node, avoiding excessive ineffective searches, and precisely guiding the search towards the target point. In conclusion, the A* algorithm incorporating the direction-guided strategy demonstrates superior performance in path planning.

Based on the five experiments conducted, it can be concluded that regardless of whether the direction-guided strategy is introduced or not, the optimal path is obtained by traversing the nodes between the starting point and the destination. The path planning results satisfy the requirements of petroleum sample transportation routes. However, when the direction-guided strategy is introduced, the path planning process tends to prioritize nodes along the optimal path, and the search nodes are more biased towards the destination. This verifies that the introduction of the direction-guided strategy makes the path search process more efficient and better aligned with the application requirements of petroleum sample transportation route planning. Therefore, we can conclude that introducing the direction-guided strategy can improve the efficiency and search quality of path planning.

Based on the experimental data in Table 3, it can be observed that the A* algorithm with the direction-guided strategy for path planning consistently reduces the time by an order of magnitude compared to the A* algorithm without the direction-guided strategy in each experiment. This confirms that the improved A* algorithm with directional guidance has a stronger sense of direction in path planning search and can improve the efficiency of path planning. Therefore, we can conclude that the A* algorithm incorporating the direction-guided strategy is a more effective path planning algorithm that can better meet practical application requirements.

**Table 3:** Path search time before and after the introduction strategy.

algorithm	optimize the time before planning 10^2^/s	optimize the time after planning 10/s	optimization rate/%
I	2.140	2.574	87.97
II	1.448	3.029	79.08
III	1.225	8.420	31.27
IV	1.322	1.469	88.89
V	1.995	7.316	63.33

**Figure 10:**
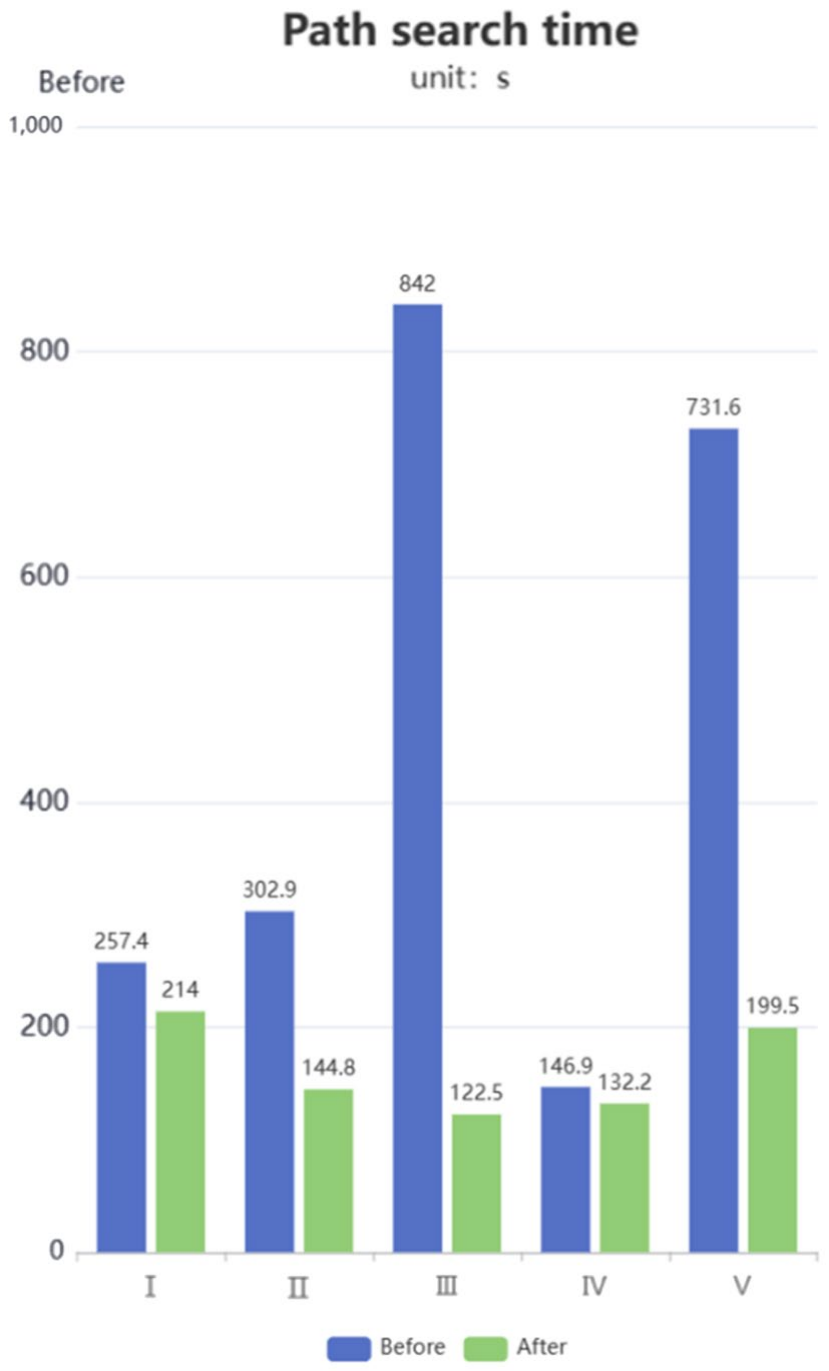
Histogram comparing the search time of paths before and after the introduction of the introduction wizard.

As shown in Fig. 10, it can be seen that after introducing the direction-guided strategy, the path planning time is shorter in each experiment compared to before optimization. Additionally, for different environments, the introduction of the direction-guided strategy can speed up path planning to varying degrees, reducing the cost of path planning in terms of time and improving the efficiency of petroleum transportation. Therefore, we can conclude that the introduction of the direction-guided strategy is an efficient method for path planning, significantly reducing path planning time and improving the efficiency of petroleum transportation.

### Comparison of search step before and after optimization

**Figure 11:**
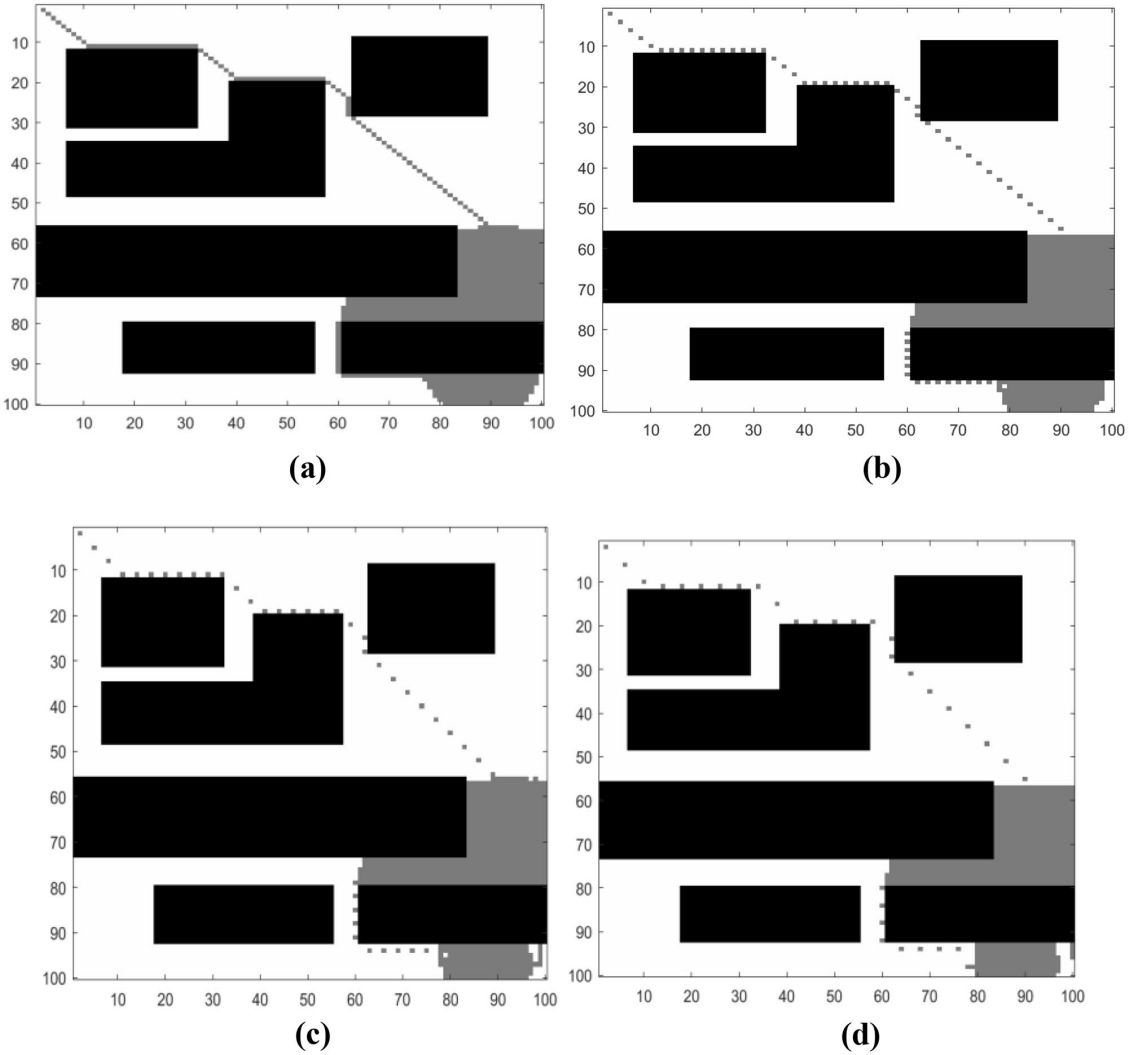
Path planning of the A* algorithm with improved step size.

Figure 11a illustrates the path planning process of the A* algorithm with continuous step size. First, the starting position is added to the Closed list. Then, starting from the starting position, the adjacent nodes around the starting point are placed in the open list. Next, for each node with a continuous step size, an evaluation is performed by calculating the heuristic estimate F = G + H. Repeat the above steps until the destination point is found. This completes the path planning process of the A* algorithm with continuous step size.

According to the experimental results of A * algorithm with one, two and three search step intervals shown in Fig. 11b–d, it can be found that when the step length is long, the number of nodes to be calculated for path planning will be reduced, and the calculation amount of A * algorithm will also be reduced. However, if the step length is too long, it may lead to the obstacle that the scale is smaller than the interval distance cannot be avoided. At this time, it is necessary to combine the local path planning dynamic window method to obtain the optimal path.

**Figure 12:**
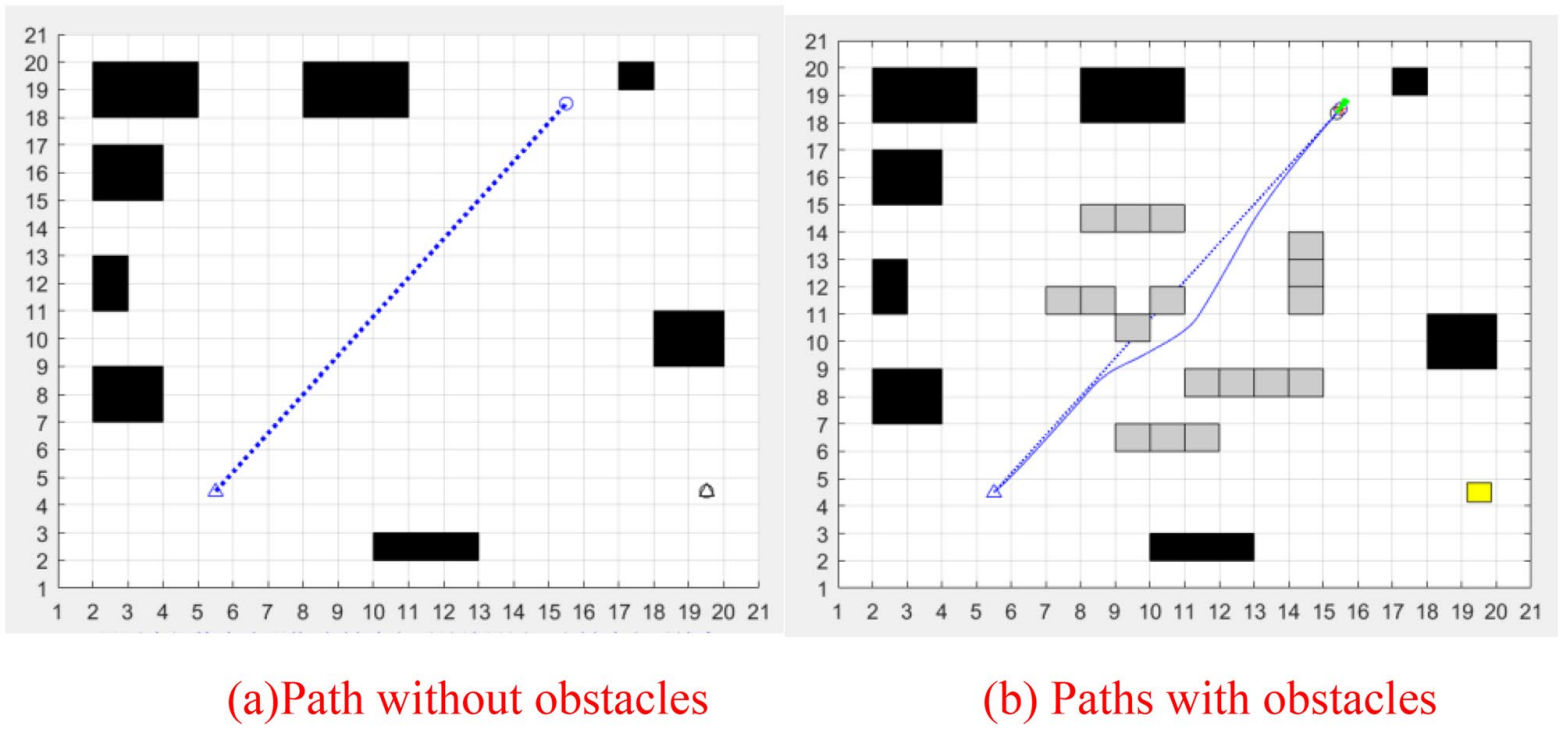
Local path planning within the step size.

As shown in Fig. 12, the local path planning simulation diagram is introduced. The black area is a fixed obstacle, the gray area is a temporary added obstacle, the small triangle is the starting point, the small circle represents the end point, the blue dotted line represents the path planned without obstacle as shown in Fig. 12a, and the blue solid line represents the path result of introducing local path planning as shown in Fig. 12b. It can be seen, it is difficult to plan the path only by using the global path planning algorithm when there are local obstacles, and good result can be achieved by using a local planning algorithm.

To sum up, in the actual path planning, the path planning step should be appropriately enlarged, and the local path planning should be combined to avoid obstacles, so as to achieve the optimal path planning effect.

RRT (Rapidly-Exploring Random Tree) algorithm is an algorithm used for robot path planning, which is suitable for solving the path planning problems under high-dimensional space and complex constraints. An experiment has been carried to compare the effect of RRT, A * algorithm and the improved A * algorithm, and the comparison results are shown in Table 4.

**Table 4:** Comparison of RRT, A* algorithm and the improved A* algorithm.

	plan time102/s	Path length102/dm	Planning efficiency
RRT	0.147	2.05	13.9
A* algorithm	2.140	9.600	4.48
Improved A*	1.028	7.754	7.59

The ratio of path length and planning time is used to reflect the efficiency of the algorithm planning path, that is, the path length planned per unit time to reflect the path planning ability of the algorithm. The greater the ratio, the higher the efficiency of path planning. Through the experimental data, it can be found that the path planning of RRT algorithm has the highest efficiency, the lowest efficiency, and the efficiency of the improved A * algorithm is higher than the original A * algorithm.

RRT algorithm is a path planning method similar to the form of tree growth structure. It is a stochastic algorithm suitable for path planning in high latitude space. The A * algorithm is determined by the enlightening function that the search results are optimal and is suitable for path planning in two-dimensional space. The sample transportation in the petrochemical plant area is based on the two-dimensional map planning path. Therefore, the path planned by choosing the improved A * algorithm is better.

## Conclusion

This article proposes an improved A* algorithm-based path planning method to enhance the transportation efficiency of oil samples. The A* algorithm is a commonly used heuristic search algorithm for graph search and path planning. It combines the characteristics of breadth-first search and greedy algorithms, allowing it to find the shortest path in a directed graph. By utilizing a heuristic function that estimates the cost from the current node to the goal node, the algorithm expands nodes in priority order from high to low until the target node is found or the entire graph is searched. An evaluation function is introduced to estimate the priority of each node. A novel evaluation function with angle constraints is proposed to achieve smoother paths. Additionally, a map-based directional guidance strategy is designed to narrow down the search range and focus the search on areas more likely to contain the optimal solution. This reduces unnecessary computations and traversals, thereby improving search efficiency. Furthermore, a search step size adjustment strategy is incorporated to allow faster convergence to the optimal solution and avoid getting trapped in local optima, saving computational resources and enhancing robustness and reliability. Simulation results demonstrate that the improved A* algorithm achieves notable improvements in path planning time, path length, and path smoothness.

In the current approach, we adjust the search step size based on the known environment to accelerate path planning. However, when dealing with an unknown environment, it becomes challenging to determine the optimal step size interval in relation to the scale of obstacles, making it difficult to determine if the path can bypass obstacles. Therefore, the path planning step size is not unique for environments with different-sized obstacles. In future research, it is hoped that this technological challenge can be overcome by dynamically adjusting the search step size in real-time based on the size of obstacles. This would enable precise improvement of path search efficiency. We also believe that with the development of artificial intelligence in the era of technology, robot path planning techniques will shine in the field of industrial development, paving the way for new avenues of technological innovation.

## Data Availability

All data generated or analyzed during this study are included in this published article.
